# Naturalistic Research on Recovery Processes: Looking to the Future

**DOI:** 10.35946/arcr.v41.1.02

**Published:** 2021-02-04

**Authors:** Robert L. Stout

**Affiliations:** Pacific Institute for Research and Evaluation, Decision Sciences Institute, Pawtucket, Rhode Island

**Keywords:** longitudinal, time-varying predictor, Bayesian decision making, behavior, alcohol

## Abstract

Because recovery is an ongoing process, conducting research on the recovery process presents multiple challenges. The process can play out over many years, but change also can occur quickly. Although researchers are keenly interested in the precursors of these sudden changes, a researcher is unlikely to be present at critical moments; however, technology offers new options not available in prior years. Recovery research at this point, however, must be pursued largely through observational methods. Experiments involving aspects of recovery can and should be done, but observation is an essential part of recovery research. Hence, this paper focuses on technologies for conducting and analyzing observational studies. The author briefly reviews methods for gathering intensive longitudinal data and discusses how recovery researchers can take advantage of existing technology to delve more deeply into the complex processes associated with recovery and relapse. The future of recovery research, however, will require examining new ways of investigating recovery phenomena, including a new option for gathering data based on decision theory. Taking maximum advantage of existing and new technology for recovery research will require increasing collaboration between recovery researchers and quantitative scientists.

## INTRODUCTION

Recovery is an ongoing process. It is ongoing both because the risk for relapse is lifelong and because renewed recovery is always possible no matter how long the relapse. The ongoing nature of recovery presents multiple research challenges. Because the process of recovery can play out over decades, longitudinal research—although often difficult to conduct—is essential. But even though the process is long, change can occur quickly.^1^ Although researchers are keenly interested in the precursors of these sudden changes, a researcher is unlikely to be present at critical moments; however, technology offers new options not available in prior years.

At this point in its scientific development, recovery research must be pursued largely through observational methods. One cannot assign research participants either to recover or to relapse at the whim of random assignment. Experiments involving aspects of recovery can and should be done, but at the current very basic stage of knowledge, observation is an essential part of recovery research. Hence, this paper focuses on technologies for conducting and analyzing observational studies. Some of these methods are familiar to addictions researchers; others, although used in other behavioral research, are not yet widely used in addictions. The processes that underlie recovery vs. relapse are exceptionally complex, which will compel us to embrace new ways to study the inner workings of these processes.

The body of the paper has three parts: (1) an overview of current technologies for gathering data on the process of recovery; (2) a review of analytical methods, including some that so far are underused; and (3) a reflection on how to move past our current approach to designing and analyzing longitudinal studies toward more quantitative, dynamic approaches. This paper does not attempt to provide an in-depth review of any of these methods, but to set the stage for a discussion of ways in which the field could develop beyond current practices.

## TECHNOLOGIES FOR GATHERING INTENSIVE LONGITUDINAL DATA

In many studies, longitudinal data have been gathered by interviews conducted at fixed intervals such as every 3 months, every 6 months, or once a year.^2^,^3^ Although this research strategy has led to some important recovery-related findings,^4^–^6^ its key limitation from the point of view of recovery research is that the use of cross-sectional data at fixed intervals risks not having adequate data on key moments of change, and it can be more challenging to characterize short-term fluctuations that may be critical in the course of recovery. For example, a client may have good support systems and be well capable of coping with anticipated challenges. But it is unlikely that support system availability is constant, and factors such as tiredness and stress may reduce the client’s ability to cope adequately with an unexpected challenge. Thus, variability over time in mediators—so far understudied—may be an important factor in recovery research.

### Calendar Recall

One way to attempt to deal with the limitations of interviews done at fixed intervals is to have study participants recall more fine-grained longitudinal data to fill in the gaps between interviews. These methods go by the generic name of calendar recall. In addictions, the most well-known of these is the Timeline Follow-Back interview for recalling alcohol consumption—and subsequently adapted for drug use—and other variables.^7^–^9^ However, these methods have been invented, apparently independently, in other fields of research including psychiatric symptomatology, notably the psychiatric status rating system developed by Keller and colleagues for Axis I disorders,^10^,^11^ and later adapted for personality disorders.^12^ Although the calendar recall method has recall and reliability limitations,^13^ and probably requires sound training and monitoring of interviewers to be fully successful,^14^ the popularity of the method across multiple studies and disciplines indicates that it continues to meet research needs.

### Ecological Momentary Assessment

Ecological Momentary Assessment (EMA) has mushroomed in popularity since first described for behavioral health audiences by Stone and Shiffman in 1994.^15^ A review of EMA methods is beyond the scope of this paper, except insofar as their implications for recovery research. In theory, EMA and related techniques offer clear advantages for recovery research in that data can be gathered during the course of participants’ daily lives, inexpensively, and close in time to the behaviors being assessed. Also, there are many options to tailor timing, prompts, and content. However, the theoretical advantages of EMA for recovery research are not always easy to achieve in practice, in particular for populations who may engage in illegal activities.^16^ The presumed benefits in terms of ecological validity may be undermined by issues such as weak compliance,^17^ reactivity from repeated measurements, and other methodological and statistical issues; see Ram et al. for an extensive discussion of threats to validity.^18^ And, considering the long-term nature of recovery, the representativeness of those study participants who are willing and able to continue engagement with an EMA protocol for an extended period is an additional issue. This is not to say that EMA studies should not be conducted with persons in recovery; as noted above, other intensive longitudinal assessment procedures have different but also serious limitations. Combining multiple methods may be useful. For example, because missed EMA reports raise the possibility of biased reporting, retrospective interviewing or specially programmed EMA probes could provide clues as to what is happening.

Although standard smartphones cannot assess blood alcohol or drug concentration, investigators have been working for many years on wearable technologies for assessing blood alcohol concentration,^19^ and some are now seeking to develop wearable sensors for at least some classes of drugs.^20^ However, these sensors continue to have technical issues that limit their accuracy, applicability, and/or device lifetime.^21^ In any case, the usefulness of wearable technologies for longitudinal research may be limited, as is the case with EMA, by issues such as selective compliance and the willingness of participants to wear them for long periods of time. The devices are likely to be most useful in short-term studies, and only after further technical development.

## DATA ANALYSIS FOR INTENSIVE LONGITUDINAL DATA

### Hierarchical Linear or Generalized Linear Modeling

Hierarchical modeling is used in situations where observations are clustered or nested; for example, researchers may wish to predict a drinking outcome at multiple points within a follow-up using measures of the frequency and/or quality of Alcoholics Anonymous participation preceding the outcome measurements. Hierarchical modeling is widely used in addictions research and is well established both for studying treatment outcome^2^,^3^ and for studying mediation of the effects of Alcoholics Anonymous.^22^,^23^ For the present purposes, the analysis will focus on the situation where time points are nested within participants. For naturalistic research on recovery where data are not necessarily gathered at fixed intervals, however, unlocking the full potential of hierarchical modeling requires a somewhat different approach than that used in treatment outcome studies. The ability of hierarchical modeling to accommodate time-varying predictor variables (often called time-varying covariates) can be helpful for studying how processes evolve over time.^24^^(ch6)^ Hierarchical modeling, often in the context of structural equation modeling, has often been used in studies of mediation.^22^,^23^,^25^ In these studies, however, assessments were usually done at fixed intervals, months apart. The rise of EMA studies and other intensive longitudinal studies, however, presents both new challenges and new opportunities to apply hierarchical methods. In particular, the number of repeated measurements can be much larger, and both missing data and designed irregular spacing of assessments make it difficult to apply the methods that have been successful in fixed-interval studies. However, hierarchical linear or generalized linear models can be used in ways that do not necessarily require predictors to be measured at fixed intervals. When missing values or irregular measurements are present, some investigators use the most recent, or most recent within a fixed window, measurement of the predictor value. This approach assumes that every predictor observation within the specified window is approximately equally strong in predicting the outcome, an assumption that, in at least some studies, can and should be tested.

### Event History Analysis

One factor that separates recovery research from outcome research is the focus of recovery research on the history of individuals. That history frequently involves major events, both negative and positive.^1^,^26^ Event history analysis historically has been largely about studying the predictors of one-time events such as death. Although there is a long history of using event history analysis in addiction,^27^ and many applications since,^28^,^29^ there are ways of extending event history models that can be advantageous for recovery researchers. Advances in event history modeling include hierarchical models for repeated events that can be useful in studying the linked processes of relapse and recovery. Like hierarchical linear modeling methods for continuous dependent variables, event history models can include time-varying predictor variables, which is especially useful for studying questions such as how the characteristics of a prior relapse affect a subsequent relapse. Studies linking onset, relapse, and recovery have appeared in the addictions literature,^30^ but useful examples also appear in the psychiatric research literature.^31^–^34^

### Graphical Methods

**Figure 1:**
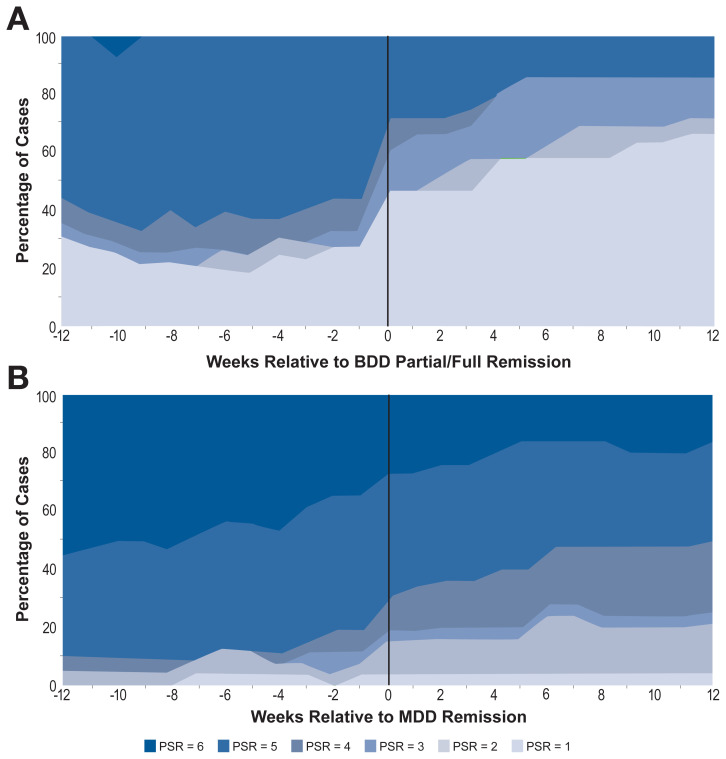
MDD PSRs over time among individuals with partial or full remission of BDD (*N* = 23) (panel A) and BDD PSRs over time among individuals with full remission of MDD (*N* = 39) (panel B) *Note:* BDD, DSM-IV body dysmorphic disorder; MDD, major depressive disorder; PSR, psychiatric status rating (psychiatric severity rating), recorded weekly, higher scores reflecting more severity, from PSR = 1, no symptoms, to PSR = 6 qualifies for full DSM-IV diagnosis. *Source:* Based on a figure from Phillips and Stout.^31^

In thinking about the role of key events in recovery, scientists are naturally interested in predicting such events. However, researchers also appreciate that both the precursors and the consequences of a major event can be complex and may play out over extended periods of time. Thus, one mission of recovery researchers is to describe quantitatively the overall course of behavior before and after a key event. For example, if depression helps lead to some relapses, does relapse occur after a sudden spike in depression, or only after a lengthy run-up? Event-locked averaging is a tool to examine such questions. Most graphs of time series data in behavioral science use a static series of time points such as baseline to month 3, month 3 to month 6, and so on. Although such graphs are useful for studying treatment outcome, it is more informative for the study of the precursors and sequelae of events to graph key variables relative to the time of an event of interest. For example, in a study of the relative course of body dysmorphic disorder (BDD) versus other Axis I disorders, the investigators examined how the severity of BDD varied before and after a participant remitted (at least 8 consecutive weeks with few or no symptoms) from major depressive disorder (MDD), and vice versa.^31^ This was a naturalistic follow-up study of 200 participants who entered the study qualifying for BDD based on criteria in the fourth edition of the American Psychiatric Association’s *Diagnostic and Statistical Manual of Mental Disorders* (DSM-IV). These participants were interviewed annually, and their clinical status was recorded on a weekly basis by using psychiatric status (clinical severity) categorical ratings; for information on the rating methodology, see Warshaw et al.^10^ and Keller et al.^11^ In the BDD study, BDD and MDD were each found to be significantly temporally related to one another.^31^ To better understand the relationship between the two disorders, event-locked graphs were created. Panel A of Figure 1 suggests that a substantial proportion of study participants who achieved full or partial remission from BDD and who had sufficient data to be included in the graph showed dramatic improvement in MDD symptoms close in time to their full or partial remission from BDD, up to and including full remission from MDD symptoms.^31^ Also, further MDD symptom recovery continued for some participants several weeks after BDD remission. (Too few participants achieved a full remission from BDD to allow a useful analysis of that group alone.) Panel B of Figure 1 shows the course of BDD symptom ratings for the 39 participants who achieved full remission from MDD. Although there was improvement in BDD symptomatology relative to MDD remission, the majority of participants continued to have high levels of BDD severity; after 12 weeks, only about 20% were at a psychiatric status rating of 2 or below, indicating few or no symptoms. These graphs tell us that the relationship between BDD and MDD is not symmetric; many with MDD recover fully whereas few with BDD do so, and the time course of change before and after the major event also differs. Although these diagrams are descriptive and must be interpreted with caution, they reveal important aspects of the time course of clinical processes around key events such as remission or relapse.

For making inferences about change in continuous or categorical outcomes before versus after an event, the method of choice is often interrupted time series analysis.^35^ In this type of analysis, it is possible to test for the presence of changes in the intercept and slope of a regression relating time to the outcome of interest. Caution must be taken, however, because the analysis must consider trends that may have existed well before the event of interest.^35^

## FUTURE DIRECTIONS

Based on the summaries above, it is evident that there is room for recovery researchers to take more advantage of existing data capture and data analysis technologies. However, ways of advancing the state of the art of recovery research also should be considered. There are two areas where further development is both needed and feasible: (1) examining the time scale of behavior change and the interplay of recovery-related variables, and (2) exploring the potential for new ways of monitoring behavior over long intervals, maximizing information capture while limiting participant burden.

### Studying the Dynamics of Behavior

Although researchers have begun to study mediators of the effect of treatment and mutual help on outcome, scant knowledge of how proposed mediators change over time unfortunately makes it difficult to design studies effectively. For example, if a popular mediator such as self-efficacy is measured 6 months after treatment and no effect has been found, would an effect have been found if the measurement had been taken at 2 months? In terms of analyzing data from an EMA study, some data on a predictor may be available from a few minutes to some days before an event of interest. How do researchers decide which of these data are “too old” to use in testing the predictor? Consider a related issue. When a predictor or a mediator assessed weeks or months before the outcome of interest is used, the implicit assumption is that the measured value of the mediator is relatively static, or that the mediator may decay after the measurement, but not before causing other changes that in turn affect outcome.

Although it is useful to do horse race comparisons of mediators,^36^ researchers must remain aware that these are static snapshot comparisons, and the importance of specific mediators may shift from within treatment to months later. Thus, researchers need to consider that behaviors, including many favorite mediators, may change over a range of time scales. For example, a mediator such as social support may build up during treatment and may fluctuate modestly as the recovering person loses old relationships and adds new ones; however, there also can be sudden major changes triggered either by the recovering person or others. Of course, in addition to studying the time scale of behavior changes, research is needed to study what variables affect the time course of mediators.

A direct way to address the need to study the time scale and predictors of change in the mediators of long-term outcome is to conduct a multivariate time series study. This would entail gathering naturalistic intensive longitudinal data (not just at two or three time points) on mediators as well as variables, such as affect and life events, that may influence the course of the mediators. As noted above, these studies are challenging, but they have been done successfully. At this stage of research, it is difficult to propose hypotheses about the relative time course of these variables, or about cross-time associations between them, so descriptive analyses may need to be employed initially.

### Making Research More Dynamic

Although branching logic and scheduled or random prompts are now common in EMA studies, they leave some problems unsolved. For example, to minimize subject burden and to be compliant with research ethics, studies allow participants to refuse to respond to prompts. Because access to participants is valuable, longitudinal studies should be designed to prioritize gathering information that is most critical to study goals, whether because of its content or because it becomes stale after a period. Writing branching logic to do this would be exceptionally difficult because of the number of combinations of circumstances that would need to be anticipated.

Decision theory offers one way to address such challenges. The most well-known approaches to optimal decision-making^37^ start with a simple premise: If two alternative actions are being considered, A1 and A2, choose the one that optimizes expected utility. Mathematically, choose A1 if E(U(A1)) > E(U(A2)), choose randomly if E(U(A1)) = E(U(A2)), and choose A2 otherwise. Although the mathematics may seem complex, researchers make complex choices all the time that implicitly require such calculations. For example, interviewers frequently encounter participants in follow-up studies who are difficult to engage and/or who have very limited time available for research interviews. To cope with these situations, investigators often give their interviewers instructions such as: “Do whatever you can to get instruments A and B, get C if possible, and finally D and E if there is an opportunity.” Mathematically, those instructions translate as: “U(A) and U(B) strongly dominate U(C), which in turn dominates U(D) and U(E), which are approximately equal.”

Decision support methods exist to support clinical investigators in estimating utility values of adequate quality to guide an automated process.^38^ The goal of that process would be to provide the necessary data to allow an EMA program to choose items in an order that reflects research priorities, much as human interviewers under pressure prioritize data to capture. A simulation study provides a simple proof of concept for this approach.^39^ It should be noted that this kind of tool for adaptive monitoring of research participants also could have treatment applications. The fact that addiction is a chronic, relapsing disorder calls out for efficient, low-cost methods for keeping in touch with clients over long periods of time without requiring substantial human labor.

## CONCLUSION

Useful technologies are available to recovery researchers to conduct complex studies of behavioral patterns and to extract increasingly useful information from these studies. It is hoped that research can find ways to build and strengthen collaborations between recovery investigators and quantitative scientists, both to take better advantage of existing technologies and to collaborate on developing new tools for further discoveries.
